# Antibiotics Dispensed to Privately Insured Pregnant Women with Urinary Tract Infections — United States, 2014

**DOI:** 10.15585/mmwr.mm6701a4

**Published:** 2018-01-12

**Authors:** Elizabeth C. Ailes, April D. Summers, Emmy L. Tran, Suzanne M. Gilboa, Kathryn E. Arnold, Dana Meaney-Delman, Jennita Reefhuis

**Affiliations:** ^1^Division of Congenital and Developmental Disorders, National Center on Birth Defects and Developmental Disabilities, CDC; ^2^Oak Ridge Institute for Science and Education (ORISE) fellowship; ^3^Office of the Director, National Center for Emerging and Zoonotic Infectious Diseases, CDC.

Urinary tract infections (UTIs) occur in about 8% of pregnant women, and untreated UTIs can have serious consequences, including pyelonephritis, preterm labor, low birth weight, and sepsis (*1*). Pregnant women are typically screened for UTIs during early pregnancy, and those with bacteriuria are treated with antibiotics (*1*,*2*). Antibiotic stewardship is critical to improving patient safety and to combating antibiotic resistance. Because of the potential risk for birth defects, including anencephaly, heart defects, and orofacial clefts, associated with use of sulfonamides and nitrofurantoin during pregnancy (*3*), a 2011 committee opinion from the American College of Obstetricians and Gynecologists (ACOG) recommended that sulfonamides and nitrofurantoin may be prescribed in the first trimester of pregnancy only when other antimicrobial therapies are deemed clinically inappropriate (*4*). To assess the effects of these recommendations, CDC analyzed the Truven Health MarketScan Commercial Database* to examine antibiotic prescriptions filled by pregnant women with UTIs. Among 482,917 pregnancies in 2014, 7.2% of women had an outpatient UTI diagnosis during the 90 days before the date of last menstrual period (LMP) or during pregnancy. Among pregnant women with UTIs, the most frequently prescribed antibiotics during the first trimester were nitrofurantoin, ciprofloxacin, cephalexin, and trimethoprim-sulfamethoxazole. Given the potential risks associated with use of some of these antibiotics in early pregnancy and the potential for unrecognized pregnancy, women’s health care providers should be familiar with the ACOG recommendations and consider the possibility of early pregnancy when treating women of reproductive age.

The MarketScan Commercial Database includes a convenience sample of employed persons with private employer-sponsored insurance and their dependents. An algorithm using insurance claims data has been developed to identify pregnant women and estimate critical periods during pregnancy (*5*). For the current analysis, CDC used the most recently available data (2013–2015) to identify pregnancies among women aged 15–44 years with an estimated LMP or date of delivery/end of pregnancy in 2014 (i.e., pregnancies that included at least one day of 2014) that ended in live birth or pregnancy loss. To capture all relevant UTI diagnosis codes and antibiotic prescriptions, the analysis was restricted to pregnant women who were continuously enrolled, or missing only one month of enrollment from 90 days before LMP to the end of pregnancy, on a health insurance plan with prescription drug coverage. Claims from physician office, urgent care, emergency department, and other outpatient visits of pregnant women were examined to identify those with a diagnosis of a UTI from 90 days before LMP through the end of pregnancy (hereafter referred to as ‘outpatient UTIs’); diagnoses associated with laboratory claims without a clinic visit were excluded. UTIs were defined as presence of an *International Classification of Diseases, Ninth Revision, Clinical Modification* (ICD-9 CM) diagnosis code of UTI (599.0) or acute cystitis (595.0 or 595.9) on at least one outpatient visit claim (*6*,*7*). Inpatient hospitalizations on the day of or day after the outpatient UTI were excluded, because these women were unlikely to have an outpatient prescription. Women with evidence of recurrent UTIs (defined as three or more UTIs from 90 days before LMP to the end of pregnancy) were also excluded, as they are likely to represent a different population from women with sporadic UTIs. For pregnant women with a UTI diagnosis claim, outpatient pharmacy claims from 2013 to 2015 were searched to identify antibiotic medications dispensed on the day of and up to 7 days after the outpatient UTI claim. The first prescription filled was used to capture the initial treatment for the UTI. If more than one antibiotic prescription was filled on the same day as the first prescription, both prescriptions were included. However, any antibiotic prescriptions filled on subsequent days were excluded. The frequency of outpatient UTIs before and during pregnancy, and the frequency, type, and timing of antibiotics dispensed were calculated. Analyses were conducted using statistical software.

Among 680,988 pregnancies in 2014 identified in the 2013–2015 data, 482,917 were eligible for further analysis (Figure 1). Among these, 34,864 (7.2%) pregnant women had an initial outpatient UTI claim 90 days before or during pregnancy. UTI diagnoses were most frequent during the first trimester of pregnancy (41.0% of UTIs) and least frequent in the third (11.8%) (Table). Overall, 68.9% of women with an outpatient UTI filled a prescription for an antibiotic within 7 days of their outpatient visit during pregnancy (median = 0 days, standard deviation = 1.1 days). In contrast, a higher proportion of women with UTIs before pregnancy filled a prescription (76.1%) during the 90 days before estimated LMP (Table). Type of antibiotic dispensed differed for UTIs treated before and during pregnancy (Figure 2). Fluoroquinolones (e.g., ciprofloxacin) and sulfonamides (e.g., trimethoprim-sulfamethoxazole) were more commonly dispensed to women within 90 days before their LMP than to pregnant women during any trimester of pregnancy. In contrast, nitrofurantoin, cephalosporins (e.g., cephalexin), and penicillins (e.g., amoxicillin) were more commonly dispensed during pregnancy than during the 90 days before LMP. The most frequently dispensed antibiotics during the first trimester of pregnancy were nitrofurantoin (34.7%), ciprofloxacin (10.5%), cephalexin (10.3%), and trimethoprim-sulfamethoxazole (7.6%) (Table).

**FIGURE 1:**
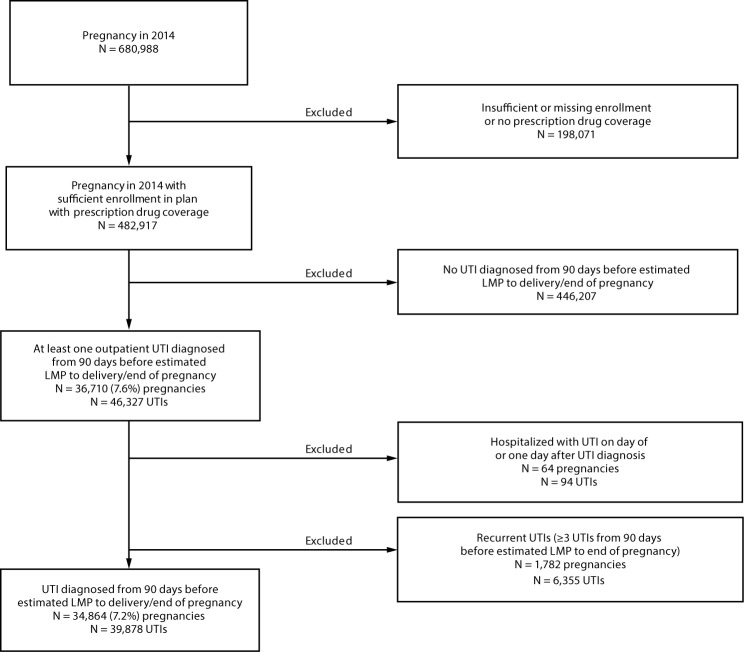
Selection of study sample of women with pregnancies in 2014 who had sufficient enrollment in a plan with prescription drug coverage* and had an outpatient claim for at least one urinary tract infection (UTI) diagnosis^†^ — Truven Health MarketScan Commercial Database, United States, 2013–2015 **Abbreviation:** LMP = date of last menstrual period. * Sufficient enrollment was defined as continuous enrollment from 3 months before date of LMP through the end of pregnancy or missing only 1 month during that period. All others were considered to have insufficient enrollment. ^†^ UTIs were defined as presence of an *International Classification of Diseases, Ninth Revision, Clinical Modification* (ICD-9 CM) diagnosis code of UTI (599.0) or acute cystitis (595.0 or 595.9) on at least one outpatient claim.

**TABLE:** Number and proportion* of women with pregnancies in 2014 who had an outpatient claim for at least one urinary tract infection (UTI) diagnosis^†^ who filled at least one prescription for an antibiotic from an outpatient pharmacy within seven days of their UTI diagnosis^§^ before or during pregnancy — Truven Health MarketScan Commercial Database, United States, 2013–2015

Medication	Period, no. (%)					
	90 days before LMP to LMP	First trimester^¶^	Second trimester^¶^	Third trimester^¶^	Any time during pregnancy	90 days before LMP through the end of pregnancy
**Total pregnancies with UTIs**	**10,864**	**14,286**	**7,880**	**4,101**	**25,264**	**34,864**
**Any antibiotic**	8,264 (76.1)	9,846 (68.9)	5,365 (68.1)	2,678 (65.3)	17,399 (68.9)	24,970 (71.6)
**Fluoroquinolones**	2,927 (26.9)	1,577 (11.0)	138 (1.8)	28 (0.7)	1,742 (6.9)	4,630 (13.3)
Ciprofloxacin	2,768 (25.5)	1,493 (10.5)	126 (1.6)	26 (0.6)	1,644 (6.5)	4,382 (12.6)
Levofloxacin	165 (1.5)	86 (0.6)	12 (0.2)	2 (0.1)	100 (0.4)	262 (0.8)
**Nitrofurantoin**	2,604 (24.0)	4,954 (34.7)	3,338 (42.4)	1,639 (40.0)	9,767 (38.7)	12,283 (35.2)
**Trimethoprim-Sulfamethoxazole**	2,031 (18.7)	1,083 (7.6)	149 (1.9)	73 (1.8)	1,304 (5.2)	3,316 (9.5)
**Cephalosporins**	560 (5.2)	1,675 (11.7)	1,216 (15.4)	659 (16.1)	3,521 (13.9)	4,062 (11.7)
Cephalexin	445 (4.1)	1,469 (10.3)	1,064 (13.5)	577 (14.1)	3,088 (12.2)	3,519 (10.1)
Cefuroxime	57 (0.5)	89 (0.6)	69 (0.9)	39 (1.0)	196 (0.8)	253 (0.7)
Cefdinir	32 (0.3)	76 (0.5)	60 (0.8)	30 (0.7)	165 (0.7)	197 (0.6)
**Penicillins**	276 (2.5)	686 (4.8)	469 (6.0)	272 (6.6)	1,416 (5.6)	1,689 (4.8)
Amoxicillin**	248 (2.3)	618 (4.3)	412 (5.2)	231 (5.6)	1,254 (5.0)	1,499 (4.3)
Ampicillin	17 (0.2)	63 (0.4)	47 (0.6)	39 (1.0)	146 (0.6)	163 (0.5)
**Other**	313 (2.9)	364 (2.6)	233 (3.0)	92 (2.2)	687 (2.7)	999 (2.9)
Metronidazole^††^	188 (1.7)	185 (1.3)	106 (1.4)	47 (1.2)	337 (1.3)	525 (1.5)
Azithromycin^††^	55 (0.5)	94 (0.7)	86 (1.1)	35 (0.9)	215 (0.9)	270 (0.8)
Other	159 (1.5)	151 (1.1)	83 (1.1)	30 (0.7)	263 (1.0)	421 (1.2)

**Abbreviation:** LMP = date of last menstrual period.

* Number and proportion sum to greater than those of “any” antibiotic because some women filled a prescription for more than one type of antibiotic. Women could also have up to two UTIs during the 90 days before LMP through the end of pregnancy.

^†^ UTIs were defined as presence of an *International Classification of Diseases, Ninth Revision, Clinical Modification* (ICD-9 CM) diagnosis code of UTI (599.0) or acute cystitis (595.0 or 595.9) on at least one outpatient claim.

^§^ Defined as the first antibiotic prescription(s) filled from an outpatient pharmacy within 7 days of UTI diagnosis.

^¶^ First trimester = 0–90 days after LMP; second trimester = 91–180 days after LMP; third trimester = 181 days after LMP until end of pregnancy.

** Includes amoxicillin/clavulanate potassium.

^††^ Typically used to treat genitourinary infections.

**FIGURE 2:**
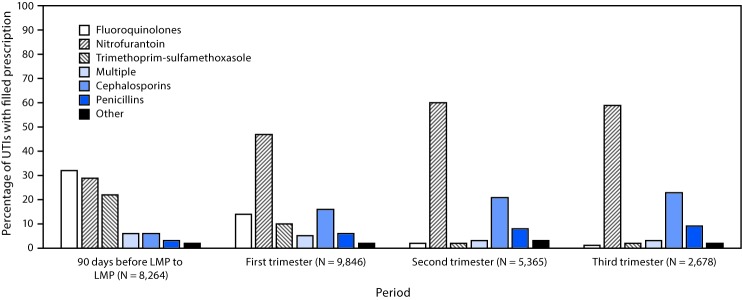
Antibiotic medication types filled from outpatient pharmacies* among women who were pregnant in 2014, had an outpatient claim for at least one urinary tract infection (UTI) diagnosis,**†** and filled a prescription for an antibiotic, by period before and during pregnancy — Truven Health MarketScan Commercial Database, United States, 2013–2015 **Abbreviation:** LMP = date of last menstrual period. * Defined as the first antibiotic prescription(s) filled from an outpatient pharmacy within 7 days of UTI diagnosis. Women with a prescription filled for more than one type of antibiotic during a given period were classified as filling prescriptions for multiple antibiotic types. ^†^ UTIs were defined as presence of an *International Classification of Diseases, Ninth Revision, Clinical Modification* (ICD-9 CM) diagnosis code of UTI (599.0) or acute cystitis (595.0 or 595.9) on at least one outpatient claim.

## Discussion

According to 2011 guidelines from the Infectious Diseases Society of America, nonpregnant women with uncomplicated UTIs should be treated with nitrofurantoin or trimethoprim-sulfamethoxazole.^†^ For pregnant women in their first trimester, a 2011 Committee Opinion from the American College of Obstetricians and Gynecologists recommended that sulfonamides and nitrofurantoin may be prescribed only if other antimicrobial therapies are deemed clinically inappropriate (*4*). In this analysis, 34.7% of pregnant women with UTIs in 2014 filled a prescription for nitrofurantoin and 7.6% filled a prescription for trimethoprim-sulfamethoxazole during their first trimester of pregnancy.

Few estimates of UTI treatment of pregnant women are available, though the current estimate is similar to a previous report of approximately 700 mothers of liveborn infants without major birth defects enrolled in a large, multisite, population-based case-control study of risk factors for major birth defects from 1997 to 2011 (*8*). In that study, approximately 6.7% of pregnant women reported at least one UTI from the month before conception through the third month of pregnancy, and two-thirds (66.6%) reported antibiotic treatment, similar to the prevalence observed in this analysis.

The current estimates of antibiotic treatment for UTIs during the 3 months before LMP are similar to estimates from previous studies of nonpregnant women. A 2003 study that examined approximately 13,000 claims among women aged 18–75 years with acute cystitis enrolled in a preferred provider care organization during 1997–1999 (*7*) found that the antibiotics most commonly dispensed within 3 days of a nonrecurrent episode of cystitis were fluoroquinolones (32%), trimethoprim-sulfamethoxazole (37%), and nitrofurantoin (16%). A recent study using the National Ambulatory Medical Care Survey and National Hospital Ambulatory Medical Care Survey to examine >7,000 outpatient visits for UTIs among women aged ≥18 years from 2002 to 2011 (*6*) found that 80% were prescribed antibiotics within 7 days of diagnosis; the most commonly prescribed medications were fluoroquinolones (39%), sulfonamides (22%), and nitrofurantoin (15%). By comparison, in the current analysis, women with UTIs during the 3 months before LMP were most often dispensed ciprofloxacin (25.5%), nitrofurantoin (24.0%), and trimethoprim-sulfamethoxazole (18.7%).

The findings in this report are subject to at least five limitations. First, pregnancies and UTI diagnoses were identified based on diagnosis and procedure codes; LMP dates, delivery dates, and UTI diagnoses were not validated (*5*). Thus, misclassification could have occurred with respect to the length of gestation, type of infection, the occurrence or timing of UTIs, and dispensing of antibiotics. Some women might have also had concomitant infections, potentially affecting the type of antibiotic prescribed. Second, pregnancies might not have been recognized by the provider or the patient at the time of UTI diagnosis and treatment. Third, these data did not allow identification of clinically appropriate nitrofurantoin or trimethoprim-sulfamethoxazole treatment that was based on urine culture or antibiotic testing. Fourth, the MarketScan Commercial Database is a convenience sample and is not generalizable to the U.S. population. Finally, antibiotic prescriptions paid for out-of-pocket were not included.

CDC’s analysis of a large insurance claims database demonstrated that, in 2014, nitrofurantoin and trimethoprim-sulfamethoxazole were common treatments for women with UTIs during their first trimester of pregnancy. Improving antibiotic selection is an important aspect of antibiotic stewardship and these antibiotics have potential risks associated with early pregnancy use, particularly during organogenesis (*3*,*8*,*9*). Given the recommendations to avoid these medications in early pregnancy if possible and the fact that nearly 50% of pregnancies in the United States are unintended (*10*), it is important that health care providers of various specialties be aware of these recommendations and that they might be “treating for two”^§^ when prescribing antibiotic treatments for UTIs to pregnant women and women who might become pregnant in the near future.

**Summary**

**What is already known about this topic?**

Because of the potential risk for birth defects, a 2011 committee opinion from the American College of Obstetricians and Gynecologists recommended that sulfonamide antibiotics and nitrofurantoin may be prescribed in the first trimester of pregnancy only when other antimicrobial therapies are deemed clinically inappropriate.

**What is added by this report?**

Nitrofurantoin and trimethoprim-sulfamethoxazole are commonly prescribed and dispensed to women with urinary tract infections during their first trimester of pregnancy.

**What are the implications for public health practice?**

Given the recommendations to avoid nitrofurantoin and trimethoprim-sulfamethoxazole in early pregnancy if possible, it is important that health care providers of various specialties be familiar with these recommendations and that they consider that they might be “treating for two” when prescribing antibiotic treatments for urinary tract infections to pregnant women and women who might become pregnant in the near future.

## Footnotes

* Truven Health Analytics, Atlanta, Georgia.

† https://academic.oup.com/cid/article/52/5/e103/388285.

§ CDC’s Treating for Two: Safer Medication Use in Pregnancy initiative aims to accelerate research on medication safety during pregnancy to provide evidence-based information for health care providers to effectively weigh the risks and benefits of treatment options for reproductive-aged women who could become pregnant. https://www.cdc.gov/pregnancy/meds/treatingfortwo/.
